# Endemic Kaposi sarcoma in HIV-negative children and adolescents: an evaluation of overlapping and distinct clinical features in comparison with HIV-related disease

**DOI:** 10.1186/s13027-018-0207-4

**Published:** 2018-11-09

**Authors:** Nader Kim El-Mallawany, Jimmy Villiera, William Kamiyango, Erin C. Peckham-Gregory, Michael E. Scheurer, Carl E. Allen, Casey L. McAtee, Alejandra Legarreta, Dirk P. Dittmer, Carrie L. Kovarik, Elizabeth Y. Chiao, Stephen C. Martin, Nmazuo W. Ozuah, Parth S. Mehta, Peter N. Kazembe

**Affiliations:** 1 Baylor College of Medicine, Department of Pediatrics, Section of Hematology-Oncology, Houston, TX USA; 2 Texas Children’s Cancer and Hematology Centers, Baylor College of Medicine, Global HOPE (Hematology-Oncology Pediatric Excellence), 1102 Bates Street, Feigin Tower, Suite 1025.16, Houston, TX 77030 USA; 3 Baylor College of Medicine Children’s Foundation Malawi, Lilongwe, Malawi; 4 Kamuzu Central Hospital, Lilongwe, Malawi; 5 Lineberger Comprehensive Cancer Center, University of North Carolina, Chapel Hill, NC USA; 6 University of Pennsylvania, Philadelphia, PA USA; 7 Center for Innovation, Quality, Effectiveness and Safety (IQuESt), Michael E. DeBakey VA Medical Center, Houston, TX USA; 8 Baylor College of Medicine, Department of Medicine, Section of Infectious Diseases, Houston, TX USA

**Keywords:** Kaposi sarcoma, Human herpesvirus-8, KS, KSHV, HHV-8, Endemic, HIV-negative, Pediatric oncology, Africa, Global health

## Abstract

**Background:**

Endemic Kaposi sarcoma (KS) was first described in African children over fifty years ago, but has recently been overshadowed by HIV-related disease. We aimed to evaluate the similarities and differences between endemic HIV-negative and epidemic HIV-positive pediatric KS in a KS-associated herpesvirus-endemic region of Africa.

**Methods:**

We describe clinical characteristics of 20 HIV-negative children with endemic KS over a six-year period and compare findings with a historical control—an HIV-related pediatric KS cohort from Lilongwe, Malawi.

**Results:**

The HIV-negative endemic KS cohort was 70% male with a median age of 9.3 years. Lymph node involvement was present in 50%, hyperpigmented skin lesions in 45%, and woody edema in 40%. One patient (5%) presented with oral KS involvement and no patients presented initially with visceral KS. Significant anemia (hemoglobin < 8 g/dL) and thrombocytopenia (platelet count < 100 × 10^9^/L) were found at time of original KS diagnosis in 45 and 40% respectively. In both HIV-negative and HIV-positive cohorts, lymphadenopathy was the most common presentation, prototypical skin lesions were often absent, severe cytopenias were a common clinical feature, and treatment outcomes were similar. Patients with endemic KS demonstrated less frequent oral involvement (5% versus 29%, *P* = 0.03) and a lower proportion of patients with visceral involvement (0% versus 16%, *P* = 0.06).

**Conclusions:**

These data suggest clinical overlap between epidemiological variants. Treatment protocols for pediatric KS in sub-Saharan Africa should be devised to include both endemic HIV-negative and epidemic HIV-related disease to better define the clinical and biological comparison.

**Electronic supplementary material:**

The online version of this article (10.1186/s13027-018-0207-4) contains supplementary material, which is available to authorized users.

## Introduction

Descriptions of endemic Kaposi sarcoma (KS) of childhood originated more than fifty years ago in central and eastern Africa—decades before the emergence of HIV—offering details of the clinical characteristics and outcomes in HIV-negative African children living in regions with endemic human herpesvirus-8/KS-associated herpesvirus (KSHV) infection [1–5]. Unique features of endemic pediatric KS that were distinct from adults included frequent presentation primarily with lymph node disease, less frequent skin involvement, and a fulminant clinical course if untreated—especially for children with lymphadenopathic KS [3, 4].

Following those initial descriptions, little has been published on endemic HIV-unrelated KS [6–9]. With the alarming rise in HIV infection rates across sub-Saharan Africa over the past 25 years, the spotlight has shifted towards HIV-related KS. Increased efforts to treat pediatric HIV infection and its complications in sub-Saharan Africa have resulted in greater awareness and understanding of HIV-related KS in children [6, 7, 10–17]. Clinical patterns of HIV-related pediatric KS appear to overlap with the early observations of endemic KS from fifty years ago. The same unique features of predilection for lymph node involvement and its characteristic association with fulminant disease progression have distinguished it from KS in HIV-infected adults [11, 12, 14–16, 18].

In this brief report, we describe the clinical features and treatment outcomes of endemic pediatric KS and compare it to our previously published experience with HIV-related pediatric KS in Lilongwe, Malawi.

## Methods

Medical records of HIV-negative children and adolescents (< 18 years) with a histological or clinical diagnosis of endemic KS between January 2011 and December 2016 were reviewed. HIV status was determined based upon standard clinical procedure using rapid antibody testing and all patients underwent repeat testing for confirmation to rule-out the potential for a false-negative result. The initial ten patients also underwent qualitative HIV DNA PCR testing for additional confirmation of their HIV-negative status. Diagnostic and therapeutic algorithms have been previously described, including supportive care strategies [16]. In brief, first line chemotherapy was a combination of bleomycin and vincristine (BV); patients with relapsed/refractory disease received doxorubicin plus BV, and the third-line chemotherapy option was paclitaxel.

Patients were staged according to the Lilongwe Pediatric KS Staging Classification, a pediatric-specific staging paradigm that attempts to address the unique clinical features of childhood KS observed in KSHV-endemic regions of Africa [17, 19]. Briefly, this staging classification differentiates four distinct clinical phenotypes with contrasting disease characteristics and treatment outcomes. Stage 1 includes patients with mild (stage 1A) to moderate (stage 1B) disease limited to the skin, subcutaneous tissue, oral mucosa, and conjunctiva. Stage 2 is defined as lymphadenopathic KS, while stage 3 is characterized by the presence of woody edema. Stage 4 is the group with the worst long-term survival and includes patients with visceral disease and those with 20 or more hyperpigmented skin/oral lesions in widespread distribution [17, 19].

Data from endemic KS patients were compared to data from a previously described cohort of HIV-related pediatric KS at the same institution [16]. All statistical analyses were performed using STATA v.13.1, and *P*-values were generated using either a *k*-sample equality-of-medians test, Chi-square test, or Fisher’s exact test, as appropriate. The study was approved by the Malawi National Health Sciences Research Committee and the Baylor College of Medicine Institutional Review Board for Human Subjects; a waiver of consent was obtained due to the retrospective nature of the study.

## Results

### Clinical characteristics of endemic pediatric KS

There were 20 children (70% male) diagnosed with endemic KS between 2011 and 2016. In comparison, there were 140 new HIV-related pediatric KS diagnoses over the same six-year time period [20]. Additional file 1: Figure S1 demonstrates a comparison of the histopathological appearance of lymphadenopathic KS in both endemic and HIV-related KS. The most common clinical site of endemic KS involvement was lymph node (50%) followed by hyperpigmented skin lesions (45%) and woody edema (40%, Table 1). No patient had visceral involvement on original presentation and one (5%) presented with oral involvement. Nine (45%) patients presented with moderate-severe anemia (hemoglobin < 8 g/dL) and 8 (40%) with moderate-severe thrombocytopenia (platelet count < 100 × 10^9^/L).

**Table 1:** Clinical Characteristics of Children with Endemic HIV-Negative KS vs HIV-Related KS

Epidemiologic subtype of kaposi sarcoma	Endemic	HIV-related	*p*
Number of Patients	20	70	
Number of Females	6 (30%)	35 (50%)	0.11^a^
Median Age in years (range)	9.3 (2.0–16.3)	8.4 (1.7–17.9)	0.57^b^
Pathology Confirmation	8 (40%)	14 (20%)	0.07^a^
Clinical Site(s) of KS Involvement			
Lymph Node	10 (50%)	52 (74%)	0.03^a^
Hyperpigmented Skin Lesions	9 (45%)	42 (60%)	0.21^a^
Woody Edema	8 (40%)	17 (24%)	0.18^a^
Flesh Colored Subcutaneous Nodules	7 (35%)	23 (33%)	0.89^a^
Oral	1 (5%)	20 (29%)	0.03^c^
Lymph Node ONLY	5 (25%)	18 (26%)	0.95^a^
Visceral	0	11 (16%)	0.06^c^
Disseminated (ie ≥20) Hyperpigmented Skin/Oral Lesions	4 (20%)	7 (10%)	0.26^c^
Anemia (hemoglobin < 8) at KS diagnosis	9 (45%)	25 (37%) *	0.54^a^
Thrombocytopenia (platelet count < 100) at KS diagnosis	8 (40%)	19 (28%) *	0.32^a^
Treatment Outcome			
Alive in Complete Remission	9 (45%)	33 (47%)	0.31^c^
Alive with Stable Disease	4 (20%)	6 (9%)	
Death	7 (35%)	31 (44%)	
Lilongwe Pediatric KS Staging Classification*			
Stage 1A (mild cutaneous/oral KS)	0	0	0.08^c^
Stage 1B (moderate cutaneous/oral KS)	4 (20%)	3 (4%)	
Stage 2 (lymphadenopathic KS)	6 (30%)	34 (50%)	
Stage 3 (woody edema KS)	6 (30%)	14 (21%)	
Stage 4 (visceral and/or disseminated KS)	4 (20%)	17 (25%)	

Legend: *KS* Kaposi sarcoma, * in the HIV-related KS cohort, 67 patients had baseline blood count assessments and 68 patients were fully staged.^a^*P*-value estimated using the Chi-square test;^b^Chi-square *P*-value estimated from the k-sample equality of medians test;^c^*P*-value estimated using the Fisher’s exact test

At a median follow-up of 39 months (interquartile range 27–62), 13 patients (65%) were alive. Nine (45%) were in complete remission (CR, defined as complete and sustained disappearance of all KS lesions), 3 (15%) were alive with stable disease, and 1 patient had experienced relapse and was receiving salvage chemotherapy. Seven (35%) patients died at median time of 2 months (range 0.1–52) from the date of KS diagnosis. Six of those deaths were attributed to KS and there were no treatment-related deaths.

### Comparison of endemic and HIV-related pediatric KS

Comparing this series of 20 children with endemic KS to our previously published cohort of 70 children with HIV-related KS, some similarities and differences emerged (Table 1).

In both cohorts, KS lymphadenopathy was the most common presenting feature, although this occurred more frequently among HIV-related KS cases (*P* = 0.03). Approximately one-fourth of both cohorts presented with KS lymphadenopathy in the absence of prototypical skin/oral lesions or woody edema (*P* = 0.95). Hyperpigmented skin lesions were present in around half of both cohorts (*P* = 0.21). Moderate-severe anemia and/or thrombocytopenia commonly occurred in both epidemiological variants (*P* = 0.54 and *P* = 0.32, respectively).

Distinctions between endemic and HIV-related KS were demonstrated as well. Patients with endemic KS had a lower frequency of oral KS involvement (*P* = 0.03) compared to the HIV-related control group. None of the endemic KS patients originally presented with visceral disease compared to 16% of HIV-related KS patients (*P* = 0.06), and the endemic KS cohort was 70% male compared to 50% in the HIV-related KS cohort (*P* = 0.11). Ultimately, treatment outcomes were similar (*P* = 0.31).

### Treatment outcomes

Nineteen patients were initiated on the chemotherapy regimen described above, with one patient having died prior to initiation of chemotherapy. Sixteen patients completed the treatment protocol and 3 died from refractory KS that progressed despite chemotherapy. Survival in context of the Lilongwe Pediatric KS Staging Classification was explored (Table 2) [17, 19]. In summary, 8 of 10 patients (80%) with stage I/II KS were alive in CR with follow-up ranging 14–62 months from time of KS diagnosis. Among the 10 patients with stage III/IV KS, only one (10%) was alive in CR. Five (50%) patients with stage III/IV disease died from relapsed/refractory KS, while 4 (40%) were alive with stable disease.

**Table 2:** Characteristics of Children and Adolescents with Endemic KS in Context of a Pediatric-Specific Staging Classification

	Stage 1: Mild/Moderate Cutaneous/Oral KS	Stage 2: Lymphadenopathic KS	Stage 3: Woody Edema KS	Stage 4: Visceral and/or Disseminated Cutaneous/Oral KS
Number of patients, *n*	4	6	6	4
Baseline Anemia (hgb < 8), *n* (%)	0	5 (83%)	1 (17%)	3 (75%)
Baseline Thrombocytopenia (platelet count < 100), *n* (%)	1 (25%)	4 (67%)	0	3 (75%)
Alive in complete remission, *n* (%)	3 (75%)	5 (83%)	1 (17%)	0
Alive with stable disease, *n* (%)	0	0	3 (50%)	1 (25%)
Deaths, *n* (%)	1 (25%)	1 (17%)	2 (33%)	3 (75%)
KS related death	0	1	2	3
Non-KS related death	1	0	0	0
2-year EFS in HIV+ KS cohort^a^	too few patients to determine	73%	29%	0
2-year OS in HIV+ KS cohort^a^		75%	79%	12%

Legend: *KS* Kaposi sarcoma, *hgb* hemoglobin, *EFS* event-free survival, *OS* overall survival, ^a^references the published historical HIV-related pediatric KS cohort (reference number 17)

Mortality patterns were categorized in context of the pediatric-specific staging paradigm as well (Table 2). No patient fit criteria for stage 1A (mild cutaneous/oral KS). Of the 4 patients categorized as stage 1B (moderate cutaneous/oral KS), there was one death, occurring in a patient who presented concurrently with KS (diagnosed clinically with prototypical skin and conjunctival eye lesions) and histologically-confirmed Hodgkin lymphoma. This patient died of refractory and progressive lymphoma; the KS lesions had completely resolved. Among the 6 patients in stage 2 (lymphadenopathic KS), there was also one death, occurring secondary to severe cytopenias on the first day of hospitalization, before chemotherapy could be initiated.

Of the 6 patients categorized as stage 3 (woody edema KS), 1 was in CR, 3 were alive with stable disease off chemotherapy, and 2 had died. One death occurred in a child that was lost to follow-up after six months of chemotherapy and was subsequently found to have died one year later, via telephone communication. The other death occurred in a child that originally presented with woody edema, hyperpigmented skin lesions, and lymphadenopathy, eventually evolving with the second relapse to include pulmonary visceral involvement with severe serosanguineous pleural effusions. All 4 of the patients categorized as stage 4 originally presented with disseminated cutaneous/oral KS (widespread distribution of ≥20 hyperpigmented skin/oral lesions) and without visceral involvement. Three of them were refractory to chemotherapy and their underlying KS rapidly progressed (time of death ranged 0.5–2 months). One patient achieved CR with up-front intensified chemotherapy (doxorubicin plus BV), however eventually experienced relapse 34 months after the original diagnosis and was alive with stable disease but receiving salvage chemotherapy with paclitaxel at the time of data analysis.

## Discussion

This cohort of HIV-negative children and adolescents with endemic KS shows considerable overlap with HIV-related disease. Primary presentation with lymphadenopathy was a consistent clinical feature in both pediatric endemic and HIV-related KS cohorts. Additionally, in both epidemiological variants, presentation with severe cytopenias is frequent and approximately half of the patients lacked the prototypical hyperpigmented skin/oral lesions that are so often relied upon as a clinical indicator of KS. Distinctions between endemic and HIV-related pediatric KS were also observed, as we noted the uncommon occurrence of oral lesions in HIV-negative patients, as well as a lower frequency of visceral involvement in endemic KS.

These findings are consistent with previously published reports on endemic KS in Africa (Table 3). The predilection for lymph node involvement in both endemic and HIV-related KS emphasizes the importance of recognizing KS as an important disease in the differential diagnosis for children with persistent lymphadenopathy in KSHV-endemic regions of Africa. Our observation of infrequent oral involvement in endemic KS has also been previously described and ranges from 0 to 10% in published historical cohorts from across the region (Table 3). This contrasts with HIV-related pediatric KS cohorts in the same region, in which oral KS lesions have been reported in 21–58% of patients [19]. It is more challenging to confirm the true incidence of visceral disease in endemic KS because of limitations in resources required to confidently establish the diagnosis in low-income countries. Additionally, there appeared to be a male predominance in endemic KS, although similar to the comparison of visceral involvement, this failed to meet statistical significance. Historically, endemic KS cohorts ranged from 70 to 84% male in publications including at least 20 subjects, while male representation in HIV-related pediatric KS has ranged from 50 to 73% [19]. While these data, as well as the historical precedents are limited by sample size, it is plausible that subtle distinctions between the two epidemiological variants of pediatric KS in Africa exist.

**Table 3:** Comparison of Clinical Features Among Endemic HIV-Negative Pediatric KS Cohorts in Africa

Location(s)	Time Period	*n*	Age of Cohort	% Male Gender	Original Clinical Presentation with KS Involvement of:				
					Lymph Node	Skin Lesions	Woody Edema	Oral Lesions	Visceral Disease
Uganda/Tanzania ^3^	1957–65	51	median 10 years	76%	53%	53%	no mention	no mention	no mention
Uganda ^4^	1968–75	12	median 8 years	58%	83%	17%	no mention	8%	8%
Tanzania ^6^	1968–82	73	63% under 5 years	84%	48%	uncertain	no mention	1%	no mention
Kenya ^7^	1997–99	8	median 7 years	88%	75%	38%	uncertain	0	13%
Blantyre, Malawi ^8^	2002–14	20	median 4 years	80%	60%	40%	70%	10%	uncertain
Lilongwe, Malawi	2011–16	20	median 9 years	70%	50%	45%	40%	5%	0

Legend: *KS* Kaposi sarcoma, no mention = indicates that these data were not mentioned at all in the publication, uncertain = indicates that although the topic was mentioned in the publication, discrete numbers were not reported. Associated references are listed in superscript in the first column

Reviewing HIV-related parameters of the HIV-infected pediatric KS cohort, a few important observations are noted that may explain the clinical overlap with endemic disease. Severe CD4 count suppression was infrequent, observed in only 28% of the HIV-related cohort [16]. Additionally, 49% of the HIV-related cohort was already on antiretroviral therapy at the time of KS diagnosis, half of them for more than 12 months [16]. Considering the relatively high proportion of HIV-infected children developing KS despite having immunocompetent CD4 counts and/or on antiretroviral therapy, it appears that the immunologic context in which HIV-related and endemic KS occurs is often similar, and potentially more attributed to an undefined qualitative immune dysfunction rather than a severe quantitative suppression of the CD4 count. The authors hypothesize that this may provide rationale for the often-overlapping clinical features observed, and may even become more apparent in the future with increasing access to antiretroviral therapy.

The occurrence of moderate-severe anemia and thrombocytopenia as a presenting clinical feature of pediatric KS has been consistent in the experiences with both HIV-negative and HIV-related disease in Malawi. Although numbers are too small in this cohort to evaluate clinical associations, presentation with cytopenias was not found to have an impact on survival in HIV-related pediatric KS [16]. Baseline cytopenias were significantly more likely to occur in HIV-infected children with stage 2 lymphadenopathic KS and stage 4 visceral and/or disseminated cutaneous/oral KS, and this pattern appears to be consistent in our experience with endemic KS as well (Table 2) [17].

Considering the frequent occurrence of cytopenias as well as the overlapping clinical patterns of disease in both epidemiological variants of pediatric KS, it is plausible that KSHV-driven pathways may play an important biological role in determining the clinical phenotype of disease. Recent pilot data from HIV-infected children in Malawi demonstrated associations between elevated plasma KSHV viral load and lymphadenopathic KS, and although sample size limited the statistical power of the results, it was notable that all 5 patients with anemia as well as all 4 patients with thrombocytopenia demonstrated detectable KSHV viral loads at the time of KS diagnosis [21]. If the clinical features are driven by KSHV-mediated pathways, they may be more attributed to mechanisms of KSHV pathogenesis, and therefore less dependent on the underlying HIV-status. Additionally, elevated interleukin-6 (IL-6) levels were common in the pilot pediatric cohort, and the links between lytic activation of KSHV, elevated viral load and IL-6 levels, and clinical presentation with cytopenias have been well established in adults with other KSHV-associated malignancies including multicentric Castleman disease (MCD) and the KSHV inflammatory cytokine syndrome (KICS) [21, 22]. Based on this, the authors hypothesize that analogous to the KSHV-driven viral pathophysiology in MCD and KICS, lytic activation of KSHV in subsets of both HIV-positive and HIV-negative pediatric KS patients may be linked with specific clinical findings (eg KS lymphadenopathy, visceral/disseminated disease, and the presence of anemia and thrombocytopenia). Ultimately, biological assessments of both epidemiological variants are required to establish the precise pathophysiological mechanisms driving the distinct clinical features that have been observed.

Cancer registry data and GLOBOCAN estimates demonstrate that alongside lymphoma (including Burkitt, Hodgkin, and non-Burkitt non-Hodgkin lymphoma), Wilms tumor, retinoblastoma, and leukemia, KS has become one of the top five most common overall childhood malignancies in several countries in eastern and central Africa including Malawi, Uganda, Kenya, Tanzania, Rwanda, Mozambique, Zambia, and Zimbabwe [23]. Contemporary pediatric KS publications from this region though, have mostly focused on HIV-infected patients. Although rates of HIV-related KS exceed those of endemic disease in this era of the HIV epidemic, it is important to establish a treatment paradigm that includes children with endemic KS as well [9]. Observations from this study suggest that overall outcomes between endemic and HIV-related cohorts are similar in the context of readily available antiretroviral therapy for HIV-infected patients. Although there are not enough patients in this analysis to draw definitive conclusions, prognosis appears to correlate more with the clinical phenotype of disease presentation (ie their classification in a pediatric-specific risk-stratification platform) rather than the underlying epidemiological classification of the disease.

Ultimately, it appears that there is significant overlap in the clinical features between endemic and HIV-related KS in children and adolescents in a KSHV-endemic region of Africa. The authors advocate for a universal risk-stratified treatment approach that applies to both endemic and HIV-related pediatric KS. Systematically characterizing the treatment response for children with endemic disease will be essential to improving overall outcomes.

## Additional file

Additional file 1: **Figure S1.** Lymph Node Histopathology in Patients with Endemic HIV-Negative and Epidemic HIV-Positive Lymphadenopathic Kaposi Sarcoma. Lymph node biopsies from endemic HIV-negative (A) and epidemic HIV-positive (B) children with lymphadenopathic Kaposi sarcoma demonstrating diffuse spindle cell tumor infiltrates that stain positive for the Kaposi sarcoma herpesvirus/human herpesvirus-8 latency-associated nuclear antigen immunohistochemical stain at 200x magnification. (TIF 7072 kb)

## Abbreviations

BV: Bleomycin and vincristine
CR: Complete remission
EFS: Event-free survival
IL-6: Interleukin-6
KICS: KSHV inflammatory cytokine syndrome
KS: Kaposi sarcoma
KSHV: Kaposi sarcoma-associated herpesvirus
MCD: Multicentric Castleman disease
OS: Overall survival
