# Supplementary figures and images for: Endemic Kaposi sarcoma in HIV-negative children and adolescents: an evaluation of overlapping and distinct clinical features in comparison with HIV-related disease
Source: Infect Agent Cancer. 2018 Nov 9;13:33. doi: 10.1186/s13027-018-0207-4 (PMC6230225; doi:10.1186/s13027-018-0207-4)

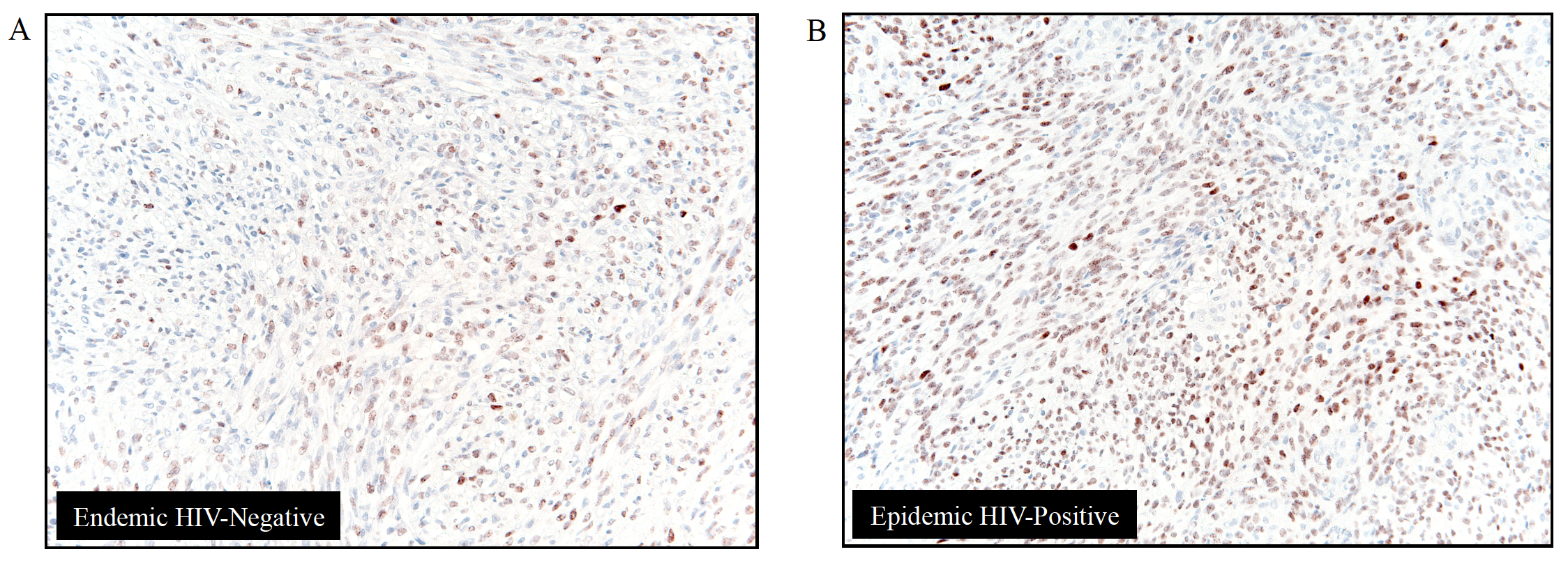

Supplement: Supplementary file 1 — Figure S1. Lymph Node Histopathology in Patients with Endemic HIV-Negative and Epidemic HIV-Positive Lymphadenopathic Kaposi Sarcoma. Lymph node biopsies from endemic HIV-negative (A) and epidemic HIV-positive (B) children with lymphadenopathic Kaposi sarcoma demonstrating diffuse spindle cell tumor infiltrates that stain positive for the Kaposi sarcoma herpesvirus/human herpesvirus-8 latency-associated nuclear antigen immunohistochemical stain at 200x magnification. (TIF 7072 kb) [file 13027_2018_207_MOESM1_ESM.tif]
